# Novel Features for Brain-Computer Interfaces

**DOI:** 10.1155/2007/82827

**Published:** 2007-08-05

**Authors:** W. L. Woon, A. Cichocki

**Affiliations:** ^1^Laboratory for Advanced Brain Signal Processing, RIKEN Brain Science Institute, 2-1 Hirosawa, Wako, Saitama 351-0198, Japan; ^2^Malaysia University of Science and Technology, Unit GL33, Block C, Kelana Square, 17 Jalan SS7/26, Petaling Jaya Selangor 47301, Malaysia

## Abstract

While conventional approaches of BCI feature extraction are based on the power spectrum, we have tried using nonlinear features for classifying BCI data. In this paper, we report our test results and findings, which indicate that the proposed method is a potentially useful addition to current feature extraction techniques.

## 1. INTRODUCTION ANDMOTIVATIONS

It is widely believed that both the underlying generators of EEG as well as the recorded signals have at least some nonlinear components [1–3]. Indeed, nonlinear tools and techniques have already been usefully deployed on problems such as the diagnosis of Alzheimer's disease [4], Schizophrenia [5], and the early prediction of epileptic seizures [6–8]. However, there appears to be a lack of interest in such methods for BCI feature extraction, which remains largely dependent on frequency-based methods. This is especially conspicuous when viewed in contrast with the enthusiastic uptake of advanced signal processing techniques for noise and artifact rejection (see, e.g., [9–11]).

To address this apparent shortcoming, we have experimented with a number of nonlinear and complexity-based feature extraction techniques. While our investigations are still preliminary, we have already obtained promising results which will hopefully encourage further progress and development in this research direction.

The rest of the paper is structured as follows. Section 2 describes the nonlinear features investigated while Section 3 explains the simulation procedures, including the data set used and preprocessing methods. The test results are presented in Section 4. Finally, Section 5 summarizes the findings and suggests possible avenues for further investigation.

## 2. EEG FEATURE EXTRACTION FOR BCI

While a variety of BCI modalities are in common use, this paper will focus on systems exploiting the modulation of *μ* (often referred to as the “sensory motor rhythm” (SMR)) and *β* rhythms [12]. Often described as attenuation of the spectral power in these bands, the associated EEG phenomena are in fact believed to be due to the desynchronization of cortical circuits related to motor function [13, 14]. From this perspective, an appropriate framework for studying these event-related EEG phenomena might be in terms of signal *complexity* .

Towards this end, some work has already been done in exploiting spatial complexity for BCI [15, 16]. However, we believe that additional information may be extracted by extending this approach to the temporal domain. As an initial review, the following measures were chosen:

1. singular spectral entropy (SSE),
2. spectral profile (SP),
3. temporal asymmetry (TA).

The above selection represents a pragmatic balance of computational simplicity and a desire to approach the nonlinear characterization problem from the complexity (SSE and SP) and statistics-based (TA) perspectives. For comparison, we also compute the averaged signal power in the *μ* (10–15 Hz) and *β*_2_ (23–28 Hz) bands, which will be referred to as the “power feature” or PF. Note that at present we only consider the high beta range (*β*_2_), as is done in a number of other studies on this data set, for example [10, 17, 18]. The exact frequency ranges mentioned above are based on the work described in [17].

These features will now be briefly described.

### 2.1. Singular spectral entropy (SSE)

If we define complexity as the number of independent though possibly interacting components which are active in a signal or system at a particular time, then one way by which complexity may be characterized is through the notion of *entropy* . While a variety of power distributions might be suitable candidates for this purpose, the shape of the singular spectrum provides an efficient representation of the constituent components of a time series. The approach chosen here is to obtain the singular spectrum of the *delay embedding* of a time series (described in further detail below), then to model this spectrum as a probability distribution before calculating the entropy of the singular values; the resulting measure will henceforth be termed the singular spectral entropy (SSE). An initial study was conducted in [19], where it was noted that imagined movements correlated with fluctuations of the SSE. Unfortunately, there was no attempt to further characterize these fluctuations or to build a classifier based on this approach. Since then, however, SSE has been applied to other aspects of EEG such as sleep [20] and ictal (seizure) EEG [6]. Hence, while its use is not widespread, SSE is a promising candidate for BCI feature extraction, motivating its use in this study.

For a time series **x**(*t*), SSE is calculated by first constructing a delay embedding matrix, **X**, of dimension *m* :

(1) $$X=[x(t),x(t+1),\ldots ,x(t+n)]\in {ℛ}^{m\times (n+1)},$$

where **x**(*t*) = [*x*(*t*),*x*(*t*−1),…*x*(*t*−*m*)]^*T*^. Next, **X** is decomposed using singular value decomposition (SVD) to obtain

(2) $$X=USV^{T},$$

where **U** and **V** are orthogonal matrices, and **S** is a diagonal matrix containing the singular values of the embedding matrix, *s*_*i*_. These are then normalized to one and used to calculate SSE as follows:

(3) $${\text{SSE}}_{m}=-\sum_{i=1}^{m}s_{i}\;\log\;s_{i}.$$

### 2.2. Spectral profile (SP)

The intuition behind the SSE feature can equally be applied to the frequency spectrum (in fact, an additional complexity measure proposed in [21] used the entropy of the power spectral density). However, experimentally we have found this measure to be extremely noisy and unsuitable for use with BCI.

However, as an alternative to SSE, we wish to define a further feature based on the shape of the power spectra; it was decided to evaluate the effectiveness of directly using the ordinates of the frequency spectra as a feature vector. To prevent the power of the signal from dominating or even affecting the classification process in any way, the extracted spectral components were first normalized to one before incorporation into the feature set. For convenience, we will refer to this feature as the *spectral profile* (SP) of the trial.

To obtain the SP vector, the elements of the spectra corresponding to the *μ* and *β* bands were extracted then normalized as follows:

(4) $$\begin{array}{ll} \mu & =\{\frac{\theta_{i}}{\sum_{j\in \Sigma_{\mu }}\theta_{j}}:i\in \Sigma_{\mu }\},\\ \beta & =\{\frac{\theta_{i}}{\sum_{i\in \Sigma_{\beta }}\theta_{j}}:j\in \Sigma_{\beta }\}, \end{array}$$

where *θ* *_i_* is the *i*th ordinate of the power spectrum, and Σ*μ* and Σ*β* are the sets of frequency ordinates falling within the two bands, respectively. ***μ*** and ***β*** are the sets containing the normalized spectral ordinates and if we define *μ* *_i_* and *β* *_j_* as the enumerated elements of these sets, the SP feature vector is then written as follows:

(5) $${\text{SP}}_{\Sigma_{\mu },\Sigma_{\beta }}=[\mu_{i},\ldots ,\mu_{N_{\mu }},\beta_{j},\ldots ,\beta_{N_{\beta }}],$$

where *N* *_μ_* and *N*_*β*_ are the sizes of sets ***μ*** and ***β***.

### 2.3. Temporal asymmetry (TA)

If we assume that the desynchronization process accompanying motor visualization reflects the activation of previously dormant neuronal circuits, then this might also be accompanied by a detectable increase in signatures of nonlinear dynamics.

One property of linear time series is that the associated statistics remain constant under time reversal, since a linear process is essentially a combination of sinusoids which are symmetric in time. This fact can be exploited to provide a particularly powerful indicator of nonlinearity, temporal asymmetry (TA); this is frequently defined as [22]

(6) $${\text{TA}}_{\tau }=\frac{\sum_{n=\tau +1}^{N}{\left(x(n)-x(n-\tau )\right)}^{3}}{{\left[\sum_{n=\tau +1}^{N}{\left(x(n)-x(n-\tau )\right)}^{2}\right]}^{3/2}},$$

where *τ* is the time delay. To restrict the analysis to components of the signal which exhibit the highest variability with respect to the classes of interest, a pair of bandpass filters was used to extract signal components in the *μ* and *β* bands (details provided later in Section 4 ), from which the TA was calculated and used to create a feature vector based on temporal asymmetry.

### 2.4. Power feature (PF)

In addition to the features mentioned above, the power feature (PF) was included for comparison. This represents the conventional approach to BCI feature extraction (variations of which are used in [10, 17, 18], e.g.) and is defined as the spectral power contained in the *μ* and *β* bands. PF is calculated, thus

(7) $$\text{PF}=[\sum_{f\in [10,15]}\theta_{f},\sum_{f\in [23,28]}\theta_{f}],$$

where *θ* *_f_* is the power spectrum at frequency *f*.

Finally, for the calculation of the SSE and TA features, we use bandpass filters to restrict the analysis to the *μ* and *β* bands of the signal, as these are known a priori to be active during motor imagery. As the actual magnitudes of the signals in these two bands are removed via normalization, we hope to focus on the overall shape of the spectrum rather than on particular peaks, as nonlinear phenomena are likely to have broader spectra compared to oscillatory generators.

## 3. PROCEDURES

### 3.1. Data

To test the proposed approach, we used dataset IIA from BCI competition 2003 [23, 24], which was provided by the Wadsworth Center, New York State Department of Health . The data consists of 64-channel recordings from three subjects (AA, BB, and CC) for ten 30-minute sessions. Each session consists of 192 trials in which the subject is required to use motor imagery to guide a cursor to one of four possible target positions. As was done during the competition, we use data from the first six sessions as the training set, while recordings of the last four sessions were used to test the trained classifiers.

### 3.2. Preprocessing and channel selections

As an initial preprocessing step, we evaluated two methods commonly used for EEG analysis: the common average reference (CAR) and the Laplacian spatial filtering methods. The CAR filter was found to significantly improve classification performance and was subsequently retained as a basic first stage in the classification process, (e.g., of its use in BCI, see [10, 18]). The CAR filter is applied as follows:

(8) $$v_{i}^{\text{CAR}}=v_{i}^{\text{Raw}}-\frac{1}{N}\sum_{j=1}^{N}v_{j}^{\text{Raw}},$$

where *N* is the number of channels in the data set, **v** ^Raw^_*i*_ is the unprocessed signal from channel number *i*, and **v** ^CAR^_*i*_ is the same channel after CAR filtering.

As CAR filtering is primarily for noise rejection, projection onto CSP (common spatial patterns) features is used to further emphasize information relevant to the BCI classification task. CSP is widely used in EEG analysis [17, 25] to find spatial filters that maximize the variance of trials corresponding to one particular class at the expense of another.

Briefly, the CSP filters are found as follows.

1. Partition the full data matrix **X** into the two class-specific matrices **X**_*A*_ and **X** *_b_* corresponding to the two classes to be discriminated.
2. Calculate the corresponding covariance matrices **C** *_A_* and **C** *_B_* as well as the sum **C** = **C**_*A*_ + **C** *_B_*.
3. Find the whitening matrix **W** such that **W** *^T^* **C** **W** = I, where **W** may be found via the eigenvector decomposition
  (9) $$C={\tilde{U}}^{T}\Sigma \tilde{U},$$
  then $\text{W}\;=\;\overset{˜}{\text{U}}\Sigma^{-1/2}$ . Hence,
  (10) $$W^{T}CW=I\Rightarrow W^{T}C_{A}W+W^{T}C_{B}W=I.$$
4. Apply a rotation matrix **Y** to both sides of (10),
  (11) $$Y^{T}(W^{T}C_{A}W+W^{T}C_{B}W)Y=I.$$
5. Choose **Y** such that it diagonalizes **W** ^*T*^ **C** *_A_* **W**, that is,
  (12) $$Y^{T}[W^{T}C_{A}W]Y=\left[\begin{array}{cccc} \sigma_{A}^{1} & 0 & \cdots & 0\\ 0 & \sigma_{A}^{2} & \cdots & 0\\ 0 & 0 & \ddots & 0\\ 0 & \cdots & 0 & \sigma_{A}^{n} \end{array}\right].$$
6. From (10)–(12), it follows that **Y** ^*T*^[**W** *^T^* **C**_*B*_ **W**]**Y** will also be diagonal, and the sum of corresponding diagonal elements will be 1.
7. Hence, to create a spatial filter that maximizes the variance of class *A* trials while minimizing the variance of class *B* trials, set **Y** to be the eigenvectors of **W** *^T^* **C** *_A_* **W**. Then, the columns of the matrix **W** **Y** provide the CSP spatial filters and may be sorted based on the eigenvalues.

For both of the data sets presented above, a bandpass filter with the following passbands: 10–15 Hz (*μ*) and 23–26 Hz (*β*) was applied. This creates two filtered signals which are then added together. As was done in [17, 18], only classes 1 and 4 are considered at this stage. Trials belonging to these two classes are extracted and combined to form **X** *_A_* and **X** *_B_*, respectively. These are then used to obtain the CSP filters as described above.

For operations requiring the power spectra, the Welch method was used to estimate the power spectral density. This method has been used in a number of other BCI related studies (e.g., [9], in which it was noted for producing superior performance). A 128-point window with 50% overlap was used. For bandpass filtering operations, we used third order Butterworth filters as they provided frequency responses with maximal flatness, (and hence minimal distortion of the amplitude spectra). The use of FIR filters was also considered for their linear phase property. However, subsequent inspections of the frequency responses revealed that a similar amplitude response would have required an FIR with around 50 taps; in comparison, the trials for subject CC are 304 samples long.

In the actual experimental setting, two CSP channels were used at any time (the actual choice of channels used varied with the subject, as will be described later). In addition, it was discovered that submitting the entire set of 64 channels to CSP processing resulted in problems with matrix singularity, as many channels are highly correlated. As such, only a subset consisting of 18 channels situated over the motor cortex was used. These were 8–10, 12–17, 19–21, 48–50, and 52–54 [18].

### 3.3. Feature translation

For feature classification, we adopt a probabilistic approach similar to that used in [26]. However, because the present data set is a lot larger, Gaussian mixture models (GMM) were used in place of the single Gaussians used in [26]. Preliminary experimentations were conducted with the case of 1 and 2 Gaussians.

To train the models, the selected features were first extracted from the training data and grouped according to target classes. For each class *c* ∈ {1, 2, 3, 4}, we train a two-Gaussian GMM using the expectation-maximization (EM) algorithm.

To classify a test vector **f** into one of the four classes, the maximum a posteriori (MAP) decision rule is used:

(13) $$c_{\text{MAP}}(f)={\text{argmax}}_{c}P(c\;|\;f).$$

*P*(*c* | **f**) can be found via Bayes' theorem. Also, in this case, we have uniform prior and constant evidence terms, hence

(14) $$\begin{array}{cc} P(c\;|\;f) & =\frac{P(f\;|\;c)p(c)}{P(f)}=kP(f\;|\;c)\\ & \propto \underset{i\in \{1,2\}}{\sum }\pi_{i,c}\exp[-{\left(f-\mu_{i,c}\right)}^{T}C_{i,c}^{-1}(f-\mu_{i,c})], \end{array}$$

where *μ*_*i*_,_*c*_ and **C**_*i*_,*_c_* are the mean and covariance of Gaussians *i* in class mixture *c*, and *π*_*i*_,_*c*_ are the mixing coefficients determined during training.

The effectiveness of the features can now be evaluated in terms of the classification rates, which are calculated as follows:

(15) $$\text{Accuracy(\%)}=100\times \frac{\left[\sum_{i=1}^{n}\delta (c_{MAP}(f_{i})-c(i))\right]}{n},$$

where *i* is the trial index and *n* is the number of trials. **f**_*i*_ and *c*(*i*) denote the feature vector and class labels for trial *i*, respectively, and *δ*(⋅) is Dirac's delta function.

## 4. RESULTS AND OBSERVATIONS

The procedures and features described in the preceding sections were applied to the BCI data and classification accuracy evaluated according to (15). For SSE, an embedding dimension of *m* = 15 was used, while for the delay parameter in TA, *τ* = 2 was used. These values were selected based on an evaluation of a range of potential combinations. The performances of all four features are compared in Tables 1 and 2 for the 1 and 2 Gaussian cases, respectively.

As can be seen, the classification performance obtained using both SSE and SP is encouraging when compared to the performance of PF. SSE in particular is more accurate than both SP and PF. SP also produced higher classification rates compared to PF though the disparity was a lot narrower. However, PF has better classification rates in the case of subject CC when compared with SP.

On the negative side, the TA feature performed very poorly. However, while disappointing, this result is not surprising considering that measures based on high-order statistics are notoriously sensitive to noise. In most cases, TA is used only in combination with surrogate data and then only to establish the presence of nonlinearity, not to characterize it.

Finally, in terms of the classification algorithm, the performance of the 1 and 2 gaussian models did not appear to differ very much. Henceforth, for brevity, we only present results produced by the two Gaussian models, which performed slightly better. However, it must be noted that the choice of either of these two models does not appear to be critical.

### 4.1. Detailed comparisons of SSE, SP, and PF

Given the disappointing classification rates obtained using TA, we exclude it from further discussions and focus now on the relative performances of SSE, SP, and PF. To better understand the performance characteristics of these three features, the per-session classification accuracies for each of the three features are presented in Table 3. For comparison, the average online accuracies (this is the success rate of the subject in hitting the target) obtained during the actual recording at the Wadsworth centre have also been included.

Some observations were as follows.

1. As mentioned before, SSE was the best all-round performer, producing the best classification rates in 7 out of 12 sessions. SP was superior to PF in 9 sessions.
2. However, SP emerged as the best feature in only 2 sessions, compared to 3 sessions in the case of PF. PF performed particularly well with subject CC, especially in session 7 where it had by far the best results. For subject BB, PF had the best classification rate for session 7 while its accuracy for session 8 was clearly better than SSE and comparable to SP.
3. Similarly, though the overall results obtained using SSE were the best, SP produced the highest average classification rate for subject BB.
4. This variability in the results implies that SSE, SP, and PF are monitoring independent aspects of the signal and that a classifier which combines the information extracted using these different features might be of value. To test this idea, we have conducted some preliminary tests, the results of which are described in Section 4.2.
5. One curious result was that the offline classification rates almost seemed to be inversely related with the online classification rates. For example, EEG recordings from subject BB, who was the highest scorer during online tests, proved to be the most difficult to analyze and resulted in the lowest offline scores. It is unclear what the cause of this inconsistency was, but we note that the same trend is observed in other studies which use this data set [10, 17, 18].
6. The choice of CSP-based spatial filter was highly dependent on the subject being tested. For EEG recordings of subjects AA and CC, CSPs specific to class 1 provided the best discrimination performance, while for subject BB, the CSPs specific to class 4 were a lot more suitable.

### 4.2. Combination classifier

Based on the variability in the results, we decided to test a combination classifier incorporating all three features {SSE, SP, PF.}

As creating a combination feature vector would greatly increase the number of parameters to be optimized, we adopted the approach used in [10], which was to train classifiers on each of the feature sets, then combine these using a committee machine. As in [10], the combined output was generated by averaging the predictions of the individual classifiers. While relatively simple, this method is acceptable as the performances of the experts do not differ too significantly.

The results of this approach are shown in Table 4. The overall impression is that the results seem to have benefited from using the combination approach. Some more detailed observations are as follows.

1. In general, the results of the combination classifier are a lot more robust compared to the results of the individual classifiers. Even though the relative performances of the three component classifiers vary quite a bit, the performance of the combination classifier either exceeds or is very close to the best of the three. This helps to confirm that the proposed features are more robust in respect to noise or variability in the data and moreover enables us to extract information which is simply not present in the power features.
2. Similarly, on a session-by-session basis, the results of the combination classifier frequently exceeds that of any of the three component classifiers. By comparing the results in Table 3 with the results in Table 4, it can be seen that in five out of twelve sessions, the combination classifier is better than all three component classifiers.
3. The classification performance of the combination classifier was comparable to results published in [10, 18]. In both cases, the combination classifier produced the best classification rate in the case of subject CC but did not fare as well in the cases of subjects AA and BB. However, the winning entry to the competition still had better performance [17], though this was obtained using a higher resolution feature extraction procedure based on dividing the trials into smaller time segments. As will be explained in Section 5 , increasing the time resolution of the proposed method is certainly one of our current objectives.
4. Similarly, the second placed entry in the competition [18] included an “energy accumulation function” to improve performance, while in [10] independent component analysis (ICA) is used to help remove noise from the signal. As a future work, we might certainly incorporate some of these enhancements but this is beyond the scope of the present paper.

## 5. DISCUSSIONS

While preliminary, the results presented here suggest that features which allow for nonlinear dynamics are promising and potentially useful in the development of BCI systems.

As the tests were conducted using offline recordings, our initial objective was not to directly compare the proposed features with existing frequency-based techniques. Because these were used as the online control signals, subjects might have been conditioned to directly modulate the power spectrum, thus biasing the results in favour of traditional approaches. As such, we did not perform extensive optimization of the feature extraction parameters; in any case, though this might have produced slight improvements to the results, it could also have resulted in overfitting or over-customization to a particular subject and was thus avoided.

Rather than obsessing with the final classification figures, our main aim was to demonstrate the general feasibility of complexity-based feature extraction. On this count, it appears that the proposed method is potentially useful for BCI. As far as we know, this is the first publication which seriously studies the performance of a temporal complexity measure on a BCI problem. If the results presented in this paper can be supported by further studies, it will provide an efficient new set of features for use with motor-imagery-based BCI systems. However, many issues need to be investigated before the practical utility of the method can be established. In particular, it should be noted that the experiments decribed in this paper process entire trials at a time to produce the classifications. While this is consistent with the approach taken in [18], a shorter-time window needs to be considered before the method can be tested for online (real-time) scenarios.

At present, we are either actively investigating or seriously considering a number of avenues for further investigation. In particular, we are interested in extracting SSE features from shorter-time windows (e.g., in the BCI experiments for this data, time windows of 200 ms were used to control the cursor motion). A separate but important issue is to find and test other practical measures of system complexity, for example, approximate entropy. If found to be promising, findings and results of these ongoing investigations will be described in a further publication in the hope of stimulating broader interest and development in this area.

## Figures and Tables

**Table 1:** Feature-wise classification accuracy using 1 Gaussian (%).

Features	Subjects			

	AA	BB	CC	Mean
SSE	68.5	52.3	68.6	63.2
SP	61.3	54.7	62.5	59.5
TA	36.8	27.9	35.0	33.2
PF	57.7	49.9	64.6	57.4

**Table 2:** Feature-wise mean classification accuracy using 2 Gaussians (%).

Features	Subjects			

	AA	BB	CC	Mean
SSE	68.4	52.3	68.7	63.1
SP	62.5	54.7	62.1	59.8
TA	36.3	29.9	35.5	33.9
PF	55.2	52.1	67.4	58.2

**Table 3:** {SSE, SP, PF}: Classification accuracy (%). Scores in bold are top scores for the respective sessions.

Features	Sessions					

	Subjects	7	8	9	10	Mean	Online
SSE	AA	**67.4**	**67.9**	**71.2**	**67.0**	68.4	73.4
	BB	52.6	52.1	**54.2**	50.5	52.3	77.2
	CC	55.4	**78.6**	**67.2**	73.6	68.7	69.0

SP	AA	65.6	62.4	68.4	53.5	62.5	73.4
	BB	58.9	**54.2**	53.1	**52.6**	54.7	77.2
	CC	53.9	67.6	60.7	66.4	62.1	69.0

PF	AA	55.2	58.9	55.7	51.0	55.2	73.4
	BB	**60.9**	52.6	43.2	51.6	52.1	77.2
	CC	**68.8**	70.8	51.6	**78.6**	67.4	69.0

**Table 4:** Combination: classification accuracy (%).

Subjects	Sessions					

	7	8	9	10	Mean	Online
AA	69.3	69.9	69.5	61.7	67.6	73.4
BB	59.9	59.4	53.1	53.6	56.5	77.2
CC	63.4	80.0	65.5	76.5	71.3	69.0
